# Comparing Cortical Bone Trajectory and Traditional Pedicle Screws in Transforaminal Lumbar Interbody Fusion: A Retrospective Cohort Study of One-Year Outcomes

**DOI:** 10.7759/cureus.43237

**Published:** 2023-08-09

**Authors:** Juanmarco Gutierrez, Andrew M Erwood, James G Malcolm, Dayton P Grogan, Alexander C Greven, Matthew F Gary, Gerald E Rodts, Geoffrey P Stricsek, Daniel Refai

**Affiliations:** 1 Neurosurgery, Emory University School of Medicine, Atlanta, USA

**Keywords:** posterior spinal instrumented fusion, lumbar spine surgery, posterior spinal fixation and fusion, pedicle screw placement, spine instrumentation

## Abstract

Introduction: This is a retrospective study of consecutive patients undergoing transforaminal lumbar interbody fusion (TLIF) at a single institution. The objective of this study was to compare the long-term results associated with cortical bone trajectory (CBT) and traditional pedicle screw (TPS) via posterolateral approach in TLIF.

Methods: Consecutive patients treated from November 2014 to March 2019 were included in the CBT TLIF group, while consecutive patients treated from October 2010 to August 2017 were included in the TPS TLIF group. Inclusion criteria comprised single-level or two-level TLIF for degenerative spondylolisthesis with stenosis and at least one year of clinical and radiographic follow-up. Variables of interest included pertinent preoperative, perioperative, and postoperative data. Non-parametric evaluation was performed using the Wilcoxon test. Fisher’s exact test was used to assess group differences for nominal data.

Results: Overall, 140 patients met the inclusion criteria; 69 patients had CBT instrumentation (mean follow-up 526 days) and 71 patients underwent instrumentation placement via TPS (mean follow-up 825 days). Examination of perioperative and postoperative outcomes demonstrate comparable results between the groups with perioperative complications, length of stay, discharge destination, surgical revision rate, and fusion rates all being similar between groups (p = 0.1; p = 0.53; p = 0.091; p = 0.61; p = 0.665, respectively).

Conclusions: CBT in the setting of TLIF offer equivalent outcomes to TPS with TLIF at both short- and long-term intervals of care.

## Introduction

Utilization of cortical bone trajectory (CBT) pedicle screws was spurred by Santoni’s contribution in 2009 describing an alternative fixation technique in patients with low bone density [1]. This seminal paper demonstrated a 30% increase in axial pullout strength relative to traditional pedicle screws (TPS) [1], and has subsequently been supported by multiple in vitro and in vivo studies [2-6].

Beyond the biomechanical benefits, CBT screws have been found to provide additional advantages in the treatment of lumbar spine pathology. A recent meta-analysis by Wang et al. compiled 14 articles from 2015-2018 comparing CBT and TPS with regard to fusion rates, functional outcomes, complication rates, revision surgery, operative time, and hospital length of stay (LOS) [7]. A comparison of 954 patients across both groups found no difference in fusion rate, visual analog scale (VAS) for back and leg pain scores, patient satisfaction or health-related quality of life (HRQOL) outcomes, and reoperation rates. CBT technique was observed to have better JOA functional improvement, shorter operative duration, lower estimated blood loss (EBL), and shorter LOS, while significantly higher (worse) Oswestry Disability Index (ODI) scores were observed in patients as well as a higher rate of adjacent segment disease [7]. Although this collection of data supports the utilization of CBT pedicle screws in the treatment of degenerative lumbar pathology, the data is heavily biased in favor of posterior lumbar interbody fusion (PLIF). Of the 14 studies, only two included transforaminal interbody fusion (TLIF); one was by the present group go uthors, Malcolm et al. and included 45 CBT TLIF patients [8], and the other was by Orita et al. and included 20 CBT TLIF patients [9].

Multiple studies have demonstrated a clear advantage of TLIF over PLIF in terms of durotomy, nerve root injury, operative duration, and transfusion rate [10-12]. The largest collection of CBT patients contained follow-up data at 90 days [8]. However, there is still a gap in the literature regarding the long-term outcomes of CBT versus TPS TLIF. The purpose of this study is to address this knowledge gap by reporting one-year postoperative outcomes of CBT TLIF in comparison to that of TPS TLIF in the largest cohort of patients represented in the literature to date.

## Materials and methods

Study design and patient selection

This was a retrospective study of consecutive patients undergoing posterior lumbar decompression with TLIF and posterior instrumentation using either TPS or CBT at Emory University Hospital Midtown, Atlanta, Georgia, United States. The study was approved by the Institutional Review Board of Emory University (approval number: IRB00094765).

Consecutive patients undergoing lumbar spine surgery with fellowship-trained neurosurgical spine faculty were identified. Inclusion criteria were: (i) TLIF for symptomatic single-level or two-level degenerative spondylolisthesis and/or lumbar stenosis, and (ii) clinical and radiographic follow-up of at least one year. Exclusion criteria were: (i) tumor, trauma, or infection as the indication for surgery; (ii) previous lumbar fusion, including anterior or posterior (history of lumbar decompression without fusion was permissible); and (iii) clinical and/or radiographic follow-up of less than one year.

Surgical technique

The surgical technique of CBT vs TPS placement was completed in accordance with previously described techniques [13]. For TPS, standard subperiosteal dissection is completed from medial to lateral to expose the spinous process, lamina, pars interarticularis, facets, and transverse process. Freehand pedicle screws are placed using intraoperative landmarks and confirmed with fluoroscopy. For CBT, subperiosteal dissection is completed from medial to lateral to expose the spinous process, lamina, pars, and facet joint to accommodate screw placement. Muscular dissection is significantly reduced. At our institution, CT guidance with intraoperative stealth navigation is used to complete CBT screw placement. Fluoroscopy can be used as an alternative. CBT follows a cauda-cephalad path in the sagittal plane and a lateral trajectory in the transverse pain, as originally described [1]. Decompression and interbody fusion were completed with standard technique [10].

Data collection

Preoperative data used for this study included age at surgery, gender, history of previous spine surgery, and baseline functional metrics such as satisfaction with current condition, VAS pain scores of the back and legs, and amount of time the patient could comfortably sit or stand. Perioperative and hospital data included pedicle screw trajectory, number of levels fused, perioperative complications, operative duration, EBL, LOS, pain medication at discharge in the form of morphine milligram equivalents (MME) per day, and discharge destination (home versus in-patient rehab as determined by a physical therapist). Post-operative data included: total follow-up based on the last clinic visit, revision surgery (when present), delayed complications, and results of imaging follow-up including lumbar spine CT scan or x-ray

Statistical analysis

Data was observed to not follow a normal distribution for age, operative duration, EBL, and MME. Non-parametric evaluation was performed using the Wilcoxon test. Fisher’s exact test was used to assess group differences for nominal data (fusion levels, complications, and revisions). Significance was established as P <0.05. Statistical analysis was completed using the JMP® Pro version 15 (2020; SAS Institute Inc., Cary, North Carolina, United States). Analysis of functional outcomes utilized an unpaired t-test at each timepoint (preopertive, six weeks, 4.5 months, and one year). GraphPad software (Insight Partners, New York City, New York, United States) was used to correct for multiple comparisons using the Holm-Sidak method.

## Results

A total of 140 patients met the inclusion criteria; 69 patients had CBT instrumentation and 71 patients underwent instrumentation placement via TPS (Table 1).

**Table 1:** Comparison of patient cohorts CBT: cortical bone trajectory; TPS: traditional pedicle screw; TLIF: transforaminal lumbar interbody fusion

	CBT	TPS	p-value
Total patients (male:female)	69 (23:46)	71 (25:45)	1.0
Average age	65	60	0.004
Previous lumbar surgery	7	7	
Single-level TLIF	57	60	
Two-level TLIF	11	5	
Two-level fusion with single-level TLIF	1	6	

CBT screws

Among the 69 CBT patients included in this study, 46 were female and 23 were male. Seven patients had previously undergone lumbar laminectomy. The average age at the time of surgery was 65 years (range 43-78 years), and the average follow-up was 17.5 months. Overall, 57 patients underwent a single-level instrumented fusion with interbody devices, 11 patients underwent a two-level instrumented fusion with interbody devices at both levels, and one patient underwent two-level fusion with interbody device at a single level. The L4-5 level accounted for 71% of interbody fusion procedures. Table 2 describes the number of interbody devices placed at each lumbar level.

**Table 2:** Comparison of levels undergoing instrumentation and fusion CBT: cortical bone trajectory; TPS: traditional pedicle screw

	CBT	TPS	p-value
			0.866
L1-2	0	1	
L2-3	0	3	
L3-4	20	9	
L4-5	57	47	
L5-S1	3	16	
All levels			0.008

Table 3 compares operative time and EBL. The average operative time for all CBT cases was 204 minutes, while for CBT instrumentation with single-level TLIF without durotomy, it was 175 minutes. The average EBL for all CBT cases was 275 mL. Sixteen patients (23%) had 21 perioperative complications; 13 patients (81%) had a durotomy, two patients (12.5%) had perioperative wound drainage which did not require surgical revision, two patients (12.5%) had urinary retention and were discharged with a Foley catheter, two patients (12.5%) had post-operative ileus, one patient (6.25%) had a urinary tract infection, and one patient (6.25%) had a pleural effusion identified on post-operative chest x-ray which did not require any intervention.

**Table 3:** Comparison of operative time and EBL CBT: cortical bone trajectory; TPS: traditional pedicle screw; EBL: estimated blood loss; TLIF: transforaminal lumbar interbody fusion

	CBT	TPS	p-value
Operative time for all cases	204	195	0.112
Operative time for single-level TLIF without durotomy (min)	175	181	0.64
EBL for all cases (mL)	275	303	0.05
EBL for single-level TLIF without durotomy (mL)	167	270	0.007

Table 4 shows discharge and follow-up data. The average LOS was 4.1 days (range 2-8 days). Data on pain medication at discharge was available for 54 patients; the average MME per day at discharge was 201. Ten patients (14.5%) were discharged to rehab and 59 (85.5%) were discharged to home. Four patients (6%) had a 30-day complication; two had superficial wound breakdown managed non-operatively and two patients underwent a wound washout (both with previous durotomy). One patient suffered wound dehiscence within the 90-day window and underwent a wound washout and revision. This was the patient’s second wound washout and this patient had a durotomy at the time of their index surgery.

**Table 4:** Comparison of discharge and follow-up data CBT: cortical bone trajectory; TPS: traditional pedicle screw; MME: morphine milligram equivalents

	CBT	TPS	p-value
Perioperative complications	21 (16 patients)	9 (9 patients)	p = 0.1
Length of stay	4.1 days	3.9 days	p = 0.53
Discharge to home	59	67	p = 0.091
Discharge to rehab	10	4	
MME	201.2	192.2	0.93
Average follow-up (days)	526	825	p = 0.004
30d Complication	4	3	
90d Complication	1	0	
Fusion rates-CT scan (%)	12 (86)	27 (96)	p = 0.665

Table 5 details revision surgeries. Seven patients (11.5%) underwent revision lumbar surgery at an average of 622 days (range 168-1082 days). Of these, four patients (57%) underwent a single-level laminectomy proximal to the level of fusion at an average of 615 days and three patients (43%) underwent revision fusion operations at an average of 630 days (53 months); one had extension of the fusion proximally, one had extension of fusion distally, and one had extension of fusion caudally with revision of previous fusion.

**Table 5:** Comparison of reoperation rates CBT: cortical bone trajectory; TPS: traditional pedicle screw

	CBT	TPS	p-value
Wound washout	3	0	
Any revision surgery	7	10	0.61
Revision proximal laminectomy	4	1	
Revision extension of fusion	2	9	
Revision of existing fusion	1	2	
Average time to revision surgery (days)	622	978	

A total of 14 patients had a post-operative CT scan at an average of 585 days after the first fusion procedure; Of these 14, 12 (86%) demonstrated solid fusion. Neither one of the two patients without solid radiographic fusion required revision surgery. The remaining 55 patients had lumbar spine x-rays available for review, all of which appeared to have mature fusion without evidence of pseudarthrosis or patient-reported low back pain.

TBS

Within the cohort receiving TBS placements, 46 were female and 25 were male. The average age at surgery was 60 years (range 41-74 years), with an average length of follow-up of 27.5 months. Seven patients had previously undergone lumbar laminectomy. Out of the 71 patients, 60 underwent a single-level instrumented fusion with interbody device, five underwent a two-level instrumented fusion with interbody devices at both levels, and six underwent a two-level instrumented fusion with interbody device at a single level. The L4-5 level accounted for 62% of interbody fusion procedures. Table 2 details the number of interbody devices placed at each lumbar level.

Average operative duration for all TPS cases was 195 minutes, and the average operative time for TPS instrumentation and single-level TLIF without durotomy was 181 minutes. The average EBL for all TPS cases was 250 mL and the average EBL for TPS instrumentation with single-level TLIF without durotomy was 238 mL. Nine patients (13%) had nine perioperative complications; one (11%) had a durotomy, three (33%) had perioperative wound drainage that did not require surgical revision, one (11%) had post-operative partial small-bowel obstruction, one (11%) had a urinary tract infection, one (11%) had a deep venous thrombosis requiring inferior vena cava filter placement, one (11%) had post-operative leg pain and was found to have a lateral pedicle screw breach on post-op CT, the leg pain resolved with steroids, and the screw was not re-positioned, and one (11%) suffered a foot drop immediately after surgery (tibialis anterior 1/5 strength postop), there was no instrumentation or implant malposition identified on post-operative imaging, and the patient did not undergo revision surgery. Fortunately, her strength improved from 1/5 to 4/5 in the follow-up.

The average length of stay was 3.9 days (range 2-9 days); four patients (5.6%) were discharged to rehab and 67 (94.4%) were discharged to home. Data on pain medications at discharge was available for 41 patients; the average MME per day at discharge was 192. Three patients (4%) had a 30-day complication: one patient (33%) was re-admitted with fevers and work-up did not identify a clear etiology, one (33%) was re-admitted with urinary tract infection (treated with antibiotics) and wound drainage treated with local wound care, and one (33%) had wound drainage treated with oral antibiotics. No patient had a 90-day complication. Ten patients (16.4%) underwent a total of 12 revision procedures at an average of 978 days (range 179-2847 days); there were two patients who each had two separate revision operations. One patient (8.3%) underwent single-level laminectomy proximal to the prior surgery, one (8.3%) underwent both proximal and distal extension of their initial fusion on separate occasions, seven (70%) underwent proximal extension of fusion, and one (16.7%) underwent two revision operations at the levels of initial surgery.

Twenty-seven patients had a post-operative CT scan at an average of 510 days after their first fusion procedure. Of these, 26 (96%) had evidence of solid fusion on CT scan, and the single patient without solid fusion had their CT scan 79 days after the initial fusion operation. The remaining 44 patients had lumbar spine x-rays available for review, all of which appeared to have mature fusion without evidence of pseudarthrosis or patient-reported low back pain.

Comparison

There was no significant difference between groups with regard to gender distribution (p = 1). CBT patients were significantly older than TPS patients (p = 0.004). Interbody fusion occurred significantly more frequently at the L4-5 level in both groups (p = 0.008), but without a significant difference when comparing TPS with CBT (p=0.866). There was no significant difference between groups when comparing the operative time for either the entire cohort (p= 0.112) or those patients undergoing single-level fusion without durotomy (p=0.64). There was a trend towards lower EBL for CBT patients; however, it did not cross the threshold for significance (p=0.5). CBT patients undergoing single-level fusion without durotomy were observed to have significantly lower EBL when compared with TPS (p = 0.007). Daily MME at discharge, perioperative complications, LOS, discharge destination, surgical revision rate, and fusion rate were similar between groups (p = 0.93; p = 0.1; p = 0.53; p = 0.091; p = 0.61; p = 0.665, respectively). See Table 3 for details.

**Figure 1:**
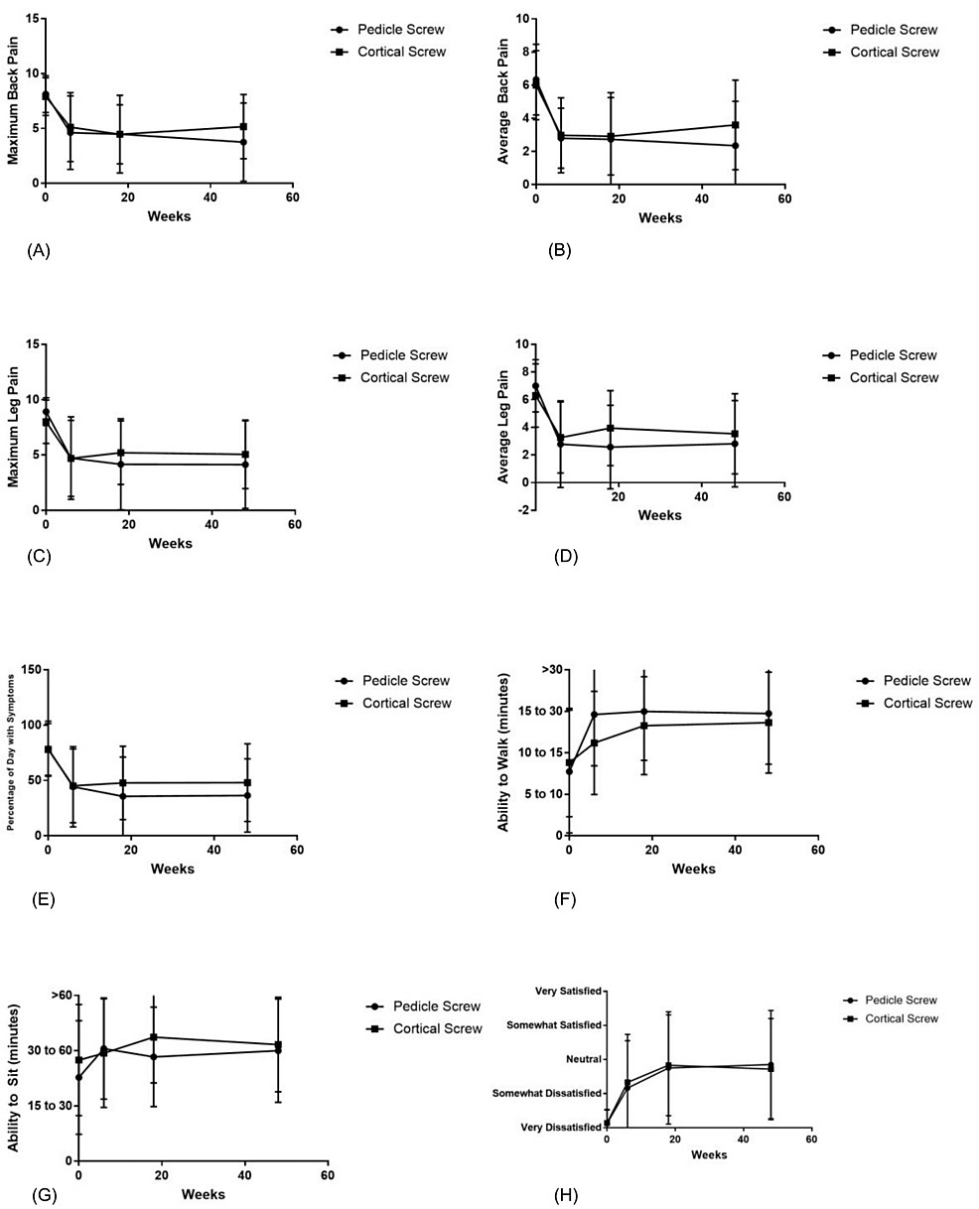
Functional outcomes between cortical bone trajectory and traditional pedicle screw patients across several measures

There were almost no significant differences in functional data when comparing groups at baseline and at one year (Figure 1). Patients in both groups had pain nearly 80% of the day prior to surgery, dropping to less than 50% of the day at one year (Figure 1A). Prior to surgery, average back pain VAS was 6 and maximum back pain VAS was 8 in both groups (Figure 1B). At one year, average back pain had dropped to 2.8 and maximum back pain was down to 3.75 in TPS patients while average back pain was 3.5 and maximum back pain was 5 in CBT patients; there was no significant difference in these scores at preoperative evaluation or one-year follow-up (p > 0.1). Preoperative average leg pain VAS was 7 and maximum leg pain VAS was 9 in TPS patients, while CBT patients had average leg pain of 6.3 and maximum leg pain of 8 (Figure 1C); TPS patients had significantly worse maximum leg pain before surgery (p = 0.031). At one-year follow-up, TPS patients had an average leg pain VAS of 2.8 and maximum leg pain of 4, CBT patients had average leg pain of 3.5 and maximum leg pain of 5; there were no significant differences in post-operative leg pain (p > 0.1) (Figure 1D). Similarly, there was no difference between groups in the percentage of day with symptoms (Figure 1E), ability to walk (Figure 1F), and ability to sit for a period of time (Figure 1G). Overall there was no difference in patient satisfaction between groups at any time point (Figure 1H).

## Discussion

CBT initially emerged as an alternative trajectory in patients with poor bone quality given its increased integration with cortical surfaces and enhanced pull-out strength [1,14]. At our institution, CBT utilization has been expanded beyond the osteoporotic patient given the benefits observed in previously published work by Malcolm [8]. This is the largest, single-series comparison of CBT pedicle screws with traditional pedicle screws in the setting of one or two-level TLIF.

Average age in the CBT group (65) was significantly higher than the TPS group (60). This was not unexpected when considering previously published work showing significantly decreased intraoperative blood loss and significantly shorter operative duration with CBT pedicle screws [8] and the need to minimize operative duration and blood loss in older patients. Interestingly, while an expanded dataset confirmed decreased blood loss when comparing CBT with TPS patients, significant differences in operative duration were not observed. Parity in operative time is, in part, attributed to our technique. At our institution, CBT pedicle screws are placed using intraoperative navigation (Medtronic Stealth; Medtronic, Minnesota, USA) with both a pre- and post-instrumentation intraoperative spin using the Medtronic O-arm (Minnesota, USA). Cumulatively, these spins can add at least 20 minutes to a case, and potentially more time, depending on radiology technologist experience. Conversely, traditional pedicle screws are placed using a limited number of intraoperative fluoroscopy images conferring minimal added operative time.

**Figure 2:**
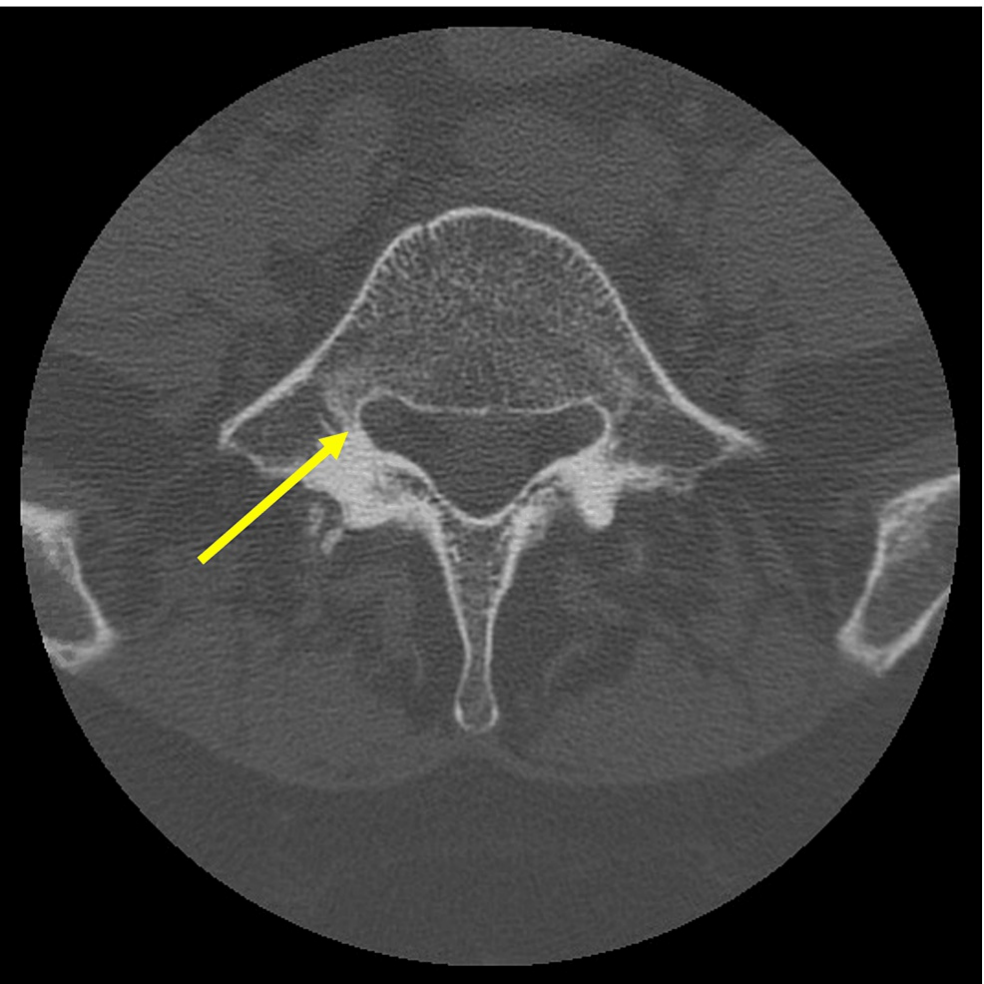
Enlarged lateral recess at L5 increases risk for medial breach with CBT pedicle screw placement. Arrow indicates lateral recess. CBT: cortical bone trajectory

There was a significant difference in the distribution of which lumbar levels underwent interbody fusion (p = 0.008), however there was no significant difference between CBT and TPS patients. While fewer CBT patients were observed to undergo interbody fusion at L5-S1 compared with TPS patients, cortical trajectory is still reasonable at this level. However, prior to planning CBT pedicle screws in a patient, it is important to consider the axial anatomy on preoperative CT imaging. Some patients have a spinal canal with a flattening and elongation of the lateral recess that narrows the corridor for the cortical trajectory and may preclude safe placement given the increased risk for medial pedicle breach (Figure 2).

Perioperative and longer-term outcomes were similar when comparing CBT and traditional trajectories. There was no significant difference in perioperative complication rate between the two groups, although the CBT group had 13 durotomies compared with the one in the TPS group. This difference in durotomy rate is likely related to the adoption of a new technique as well as the older patient age in the CBT group, as age has been observed to correlate with incidental durotomy rate [15]. Total follow-up was significantly longer in the TPS group compared with the CBT group. This was expected given the relatively novel nature of the cortical trajectory when compared with traditional trajectory. There was no significant difference in the rate of revision surgery between groups (11.5% for CBT versus 16.4% for TPS; p=0.61). Proximal laminectomy was more common in the CBT group while extension of fusion was more common in the TPS group. While relatively few patients in each group had post-operative CT scans (20% of CBT patients and 39% of TPS patients), fusion rates were not statistically significant (p = 0.665).

The functional data collected in this series were in agreement with the conclusions reached by Wang et al. [7] observing similar outcomes when comparing CBT and TPS instrumentation techniques. Collection of patient-reported metrics is not standardized within our clinic setting and varies based on provider organization and phase of care. Response rates to each functional parameter were variable even within individual patients. One year response rates for TPS patients ranged from 24-38% and 43-67% for CBT patients.

This is the largest single-institution collection of CBT patients undergoing TLIF. The data demonstrate equivalent outcomes when compared with traditional pedicle screw placement when analyzing 1-year outcomes. Cortical trajectory should be considered a safe and effective addition to the spine surgeon’s armamentarium for the treatment of one or two-level degenerative lumbar pathology.

Limitations

This is a retrospective review of data from a single center, limiting this study to level 3 evidence. While the patient pool is derived from three fellowship-trained neurosurgeons, the majority of the CBT patients were operated on by a single author and these results are not necessarily replicated by other providers. However, all procedures were also performed in an academic setting while training residents and fellows; adoption and refinement of the cortical technique by the independent practitioner is more likely to derive the benefits of improved operative efficiency observed in the earlier work by Malcom [8].

As discussed above, follow-up was significantly different between groups. All patients had at least 1-year follow-up, but given the more recent adoption of CBT pedicle screws, it is not surprising that patients with traditional pedicle screws had a significantly greater length of follow-up. It is possible that the rate of revision surgery after CBT instrumentation may rise as duration of surveillance increases. Functional data capture was also quite low (<50%) across both groups. While no significant differences were observed, the dataset was underpowered.

Post-operative CT scans were limited to only 14 of 69 CBT patients (20%) and 28 of 71 TPS patients (39%). While plain radiographs are adequate for routine follow-up, CT imaging is more accurate when evaluating fusion status and pseudarthrosis. However, post-operative CT is most often obtained to rule out symptomatic pseudarthrosis and it is likely that fusion status observed in most of the patients followed only with x-rays is appropriate.

## Conclusions

Cortical bone trajectory pedicle screws in the setting of TLIF offer equivalent outcomes to traditional pedicles screws with TLIF. Our data demonstrated the CBT technique conferred a benefit in terms of blood loss when compared with traditional pedicle screw placement. Modification of operative technique (example: robotics) could also confer a time benefit when utilizing CBT pedicle screws, however this was not analyzed in this study.
